# The Relationships Between Family Characteristics and Undergraduate Students' COVID-19 Responses: A Cross-Sectional Study in China

**DOI:** 10.3389/fpubh.2022.873696

**Published:** 2022-05-02

**Authors:** Teng Zhao, Qiang Su, Xinye Hu

**Affiliations:** ^1^Zhejiang Academy of Higher Education, Hangzhou Dianzi University, Hangzhou, China; ^2^Institute of Higher Education, Wenzhou Medical University, Wenzhou, China; ^3^Center for the Study of Higher and Postsecondary Education, University of Michigan, Ann Arbor, MI, United States

**Keywords:** COVID-19, family characteristics, disaster responses, undergraduate students, sex and ethnicity

## Abstract

The COVID-19 pandemic has dramatically threatened the post-secondary education setting. It is crucial to understand the factors that potentially affect college students' COVID-19 responses, such as risk awareness, knowledge of the disease, and pandemic preparedness. However, there is insufficient literature on whether family characteristics contribute to students' COVID-19 responses. Leveraging the data from self-administrated survey – titled College Students' Epidemic Preparedness in the Post-COVID-19 Era (CSEPPCE), we find that students from high-income families were more likely to have a greater awareness of risk and better knowledge of COVID-19. Additionally, students whose parents were employed by the government had a higher probability of knowing COVID-19 symptoms and wearing masks. However, the relationships among risk awareness, knowledge, and family income did not meaningfully vary by sex or ethnicity. Implications and future directions are discussed.

## Introduction

The COVID-19 virus dramatically threatens global public health. Though people have taken numerous actions to prevent infection, thousands of confirmed cases and deaths are still precipitously reported as new variants continue to emerge (1). This global health crisis has alerted individuals to be well-prepared for diseases, especially from the mental aspect. As one of the largest components of the population in China, ~33 million undergraduate students were seeking a postsecondary degree in public universities and colleges in 2020 (2). College students have been historically regarded as a privileged population, and postsecondary institutions are a unique setting for health promotion (3). However, the pandemic has exacerbated students' risk for mental wellbeing and has simultaneously affected their academic performance (4), putting post-secondary student success in jeopardy. Thus, it is crucial to understand the factors that potentially affect students' COVID-19 responses, such as risk awareness of the pandemic, knowledge of the disease, and pandemic preparedness.

Previous literature has found that family factors have been associated with family members' disaster reactions (5, 6). For example, Armaş et al. surveyed ~1,400 citizens on earthquake risk perception and found citizens' stress vulnerability, such as frequency of feeling nervous and stressed, were negatively associated with their family income (7). Donner and Lavariega-Montforti collected data from 740 residents in the Rio Grande Valley and concluded that income was a positive predictor for earthquake preparedness (8). As such, obstacles from storing disaster supplies were the main concern for low-income immigrant Latinos in disaster preparedness (9). It seems that family income plays a crucial role in disaster responses. According to the Bioecological Model of Human Development, Bronfenbrenner & Morris illustrated that middle-class parents were likely to have greater access to resources that could offer needed information, knowledge, and/or experience for their children (10). In Chinese society, government employees, professionals, and business owners are regarded as middle class, and their children tend to be positively affected in terms of the Bioecological Model. Thus, our study assumes that family income and parental occupations will likely influence students' responses to COVID-19. For example, wealthy families will likely have a better educational background and more resources, which will increase family members' knowledge of and economic preparedness for COVID-19.

Furthermore, families are the primary sources for adolescents aged 16–19 to receive pandemic-related information (11). As Chinese society returns to normal operations after the pandemic has been effectively controlled, the amount of information concerning the outbreak has gradually maintained a normal level in social media. According to Down's issue-attention cycle (12), at the current stage, the public's attitude toward COVID-19 has not been as severe as before. Accordingly, families are functioning critically in collecting pandemic information and alerting family members, especially when there are still intermittent outbreaks in some areas.

This study aims to understand the relationship between family characteristics and students' responses to COVID-19 under normal operation. This study contributes to the existing literature and proposes policy recommendations to inform related stakeholders, such as postsecondary administrators and leaderships, how to effectively guide students to appropriately respond to COVID-19. To the authors' knowledge, there is insufficient literature on whether family characteristics contribute to students' COVID-19 awareness, knowledge, and preparedness. This study explores these relationships by leveraging the data from self-administered questionnaire–titled College Students' Epidemic Preparedness in the Post-COVID-19 Era (CSEPPCE).

## Materials and Methods

### Data and Sample

In May 2021, we distributed the survey of CSEPPCE through the online survey platform WJX.CN to traditional full-time undergraduate students from 13 public 4-year post-secondary institutions in an eastern province in China. The survey was comprehensively designed in terms of Ahmed et al. (13) and Ikhlaq et al. (14). The survey collected students' information on their COVID-19 awareness, knowledge, and preparedness. Since Chinese universities and colleges would adopt different levels of anti-epidemic measures based on the real-time situation of the pandemic, it also gathered students' responses to campus anti-epidemic climate change to better understand the dynamic of students' responses under different situations. In addition, it asked for their family information, such as annual family income and parental occupations. This provided a unique opportunity to examine the relationships between students' family characteristics and their responses to COVID-19.

First, using the convenience sampling method, we selected 34 faculty members from 34 public 4-year postsecondary institutions, and 13 (38.24%) were willing to help distribute the survey. Second, to ensure that the representative sample has sufficient statistical power, a sample size calculator (15) was implemented to obtain a recommended sample size larger than 1,036, with a 4% margin of error, a 99% confidence level, and a 50% response distribution. This was done to represent the 2021 data of 1,148,737 undergraduate students from the Provincial Bureau of Statistics (16). Third, the 13 identified faculty members distributed the survey link to a total of 1,600 full-time undergraduate students, and obtained 1,470 responses (91.88% response rate). Since there were 22.7% missing values in students' grade point average (GPA), which was one of the intended variables for the present study, we applied multiple imputation techniques (17), yielding an analytic sample of 1,470, which consisted of 53% male and 47% female students (see Table 1).

**Table 1:** Descriptive statistics of intended variables.

		**N**	**Mean**	**SE**	**95% CI**	
					**Lower**	**Upper**
**Dependent variable**						
*COVID-19 awareness*						
	Discuss with classmates	1,470	2.37	0.02	2.33	2.41
	Discuss with friends	1,470	2.26	0.02	2.22	2.30
	Discuss with family	1,470	2.29	0.02	2.25	2.33
*COVID-19 knowledge*						
	Symptom	1,470	2.93	0.02	2.88	2.97
	Transmission	1,470	3.07	0.02	3.02	3.11
	Preventative measures	1,470	3.16	0.02	3.11	3.20
*COVID-19 preparedness*						
	Mask	1,470	2.22	0.02	2.17	2.26
	Hand sanitizer	1,470	2.25	0.02	2.20	2.29
	Social distance	1,470	2.24	0.02	2.19	2.28
*Campus climate change*						
	Perceived climate	1,470	0.89	0.01	0.87	0.90
	Mask-wearing	1,470	0.56	0.01	0.53	0.58
	Keeping social distance	1,470	0.47	0.01	0.44	0.49
	Participating in campus activities	1,470	0.42	0.01	0.39	0.44
**Independent variable**						
*Demographic characteristics*						
	Male	777	52.86%	0.01	0.50	0.55
	Female	693	47.14%	0.01	0.45	0.50
	Han	1,378	93.74%	0.01	0.93	0.95
	Other	92	6.26%	0.01	0.05	0.07
	Urban	528	35.92%	0.01	0.33	0.38
	Suburban	158	10.75%	0.01	0.09	0.12
	Rural	686	53.33%	0.01	0.51	0.56
*Family characteristics*						
	Low-income	403	27.41%	0.01	0.25	0.30
	Mid-income	1,008	68.57%	0.01	0.66	0.71
	High-income	59	4.01%	0.01	0.03	0.05
	Father's job-G	174	11.84%	0.01	0.10	0.13
	Father's job-P	414	28.16%%	0.01	0.26	0.30
	Father's job-S	323	21.97%	0.01	0.20	0.24
	Father's job-O	559	38.03%	0.01	0.36	0.41
	Mother's job-G	151	10.27%	0.01	0.09	0.12
	Mother's job-P	288	19.59%	0.01	0.18	0.22
	Mother's job-S	387	26.33%	0.01	0.24	0.29
	Mother's job-O	644	43.81%	0.01	0.41	0.46
*Academic performance*						
	College GPA	1,470	3.19	0.02	3.15	3.24

*In job category, G, government employee; P, professionals and experts; S, service-related jobs; O, other jobs*.

### Measures

#### Dependent Variables

Students' responses to COVID-19 are the dependent variables of primary interests and mainly consist of four categories: students' risk awareness of COVID-19, knowledge of COVID-19, preparedness for COVID-19, and their responses to campus anti-epidemic climate change. Each category consisted of 3–4 survey items that were measured on a 5-point Likert scale with 1 representing “least” and 5 representing “most.” In students' risk awareness of COVID-19, the following questions were asked: “how frequently will you discuss COVID-19 with your classmates,” “how frequently will you discuss COVID-19 with your friends,” and “how frequently will you discuss COVID-19 with your family.” The mean of each item was 2.37, 2.26, and 2.29, respectively. In students' knowledge of COVID-19, we surveyed “how familiar are you with COVID-19 symptoms,” “how familiar are you with COVID-19 transmission,” and “how familiar are you with COVID-19 preventative measures,” where the corresponding means were 2.93, 3.07, and 3.16, respectively. In students' preparedness for COVID-19, we collected: “how frequently will you wear a mask when going to public places,” “how frequently will you use hand sanitizer,” and “how frequently will you keep social distance when in public,” with means of 2.22, 2.525, and 2.24, respectively.

The last set of dependent variables was students' responses to campus anti-epidemic climate change. We asked: “could you perceive campus anti-epidemic climate change,” “would it affect your frequency of wearing a mask when you feel campus anti-epidemic measures are loose,” “would it affect your social distancing when you feel campus anti-epidemic measures are loose,” and “would you want to participate in campus activities when you feel campus anti-epidemic measures are loose.” From Table 1, the results show that 89% of students could perceive campus anti-epidemic climate change. When the climate is loose, 56% of students would be influenced to wear a mask, 47% of students would be influenced to remain socially distanced, and 42% of students would like to participate in campus activities.

#### Independent Variables

The primary interests of independent variables were family characteristics, including family income and parental occupation. Annual family income was divided into three categories: low income = “< 30000 CNY,” middle-income = “> 30000 CNY and < 500000 CNY,” and high income = “>500000 CNY.” According to the Occupational Classification of the People's Republic of China, the father's and mother's occupations were sorted into eight categories: government employee; professionals; clerks and related personnel; business and service personnel; production experts in agriculture, forestry, animal husbandry, fishing, and water conservancy industries; production and transportation equipment operators and related experts; soldiers; and others. For the convenience of analysis, these categories were instead recoded into four categories: government employees (including soldiers because they also work for the government), professionals and experts in all fields, service-related jobs, and other jobs.

Other than family characteristics, students' demographic characteristics, such as sex, ethnicity, and urbanicity, defined as where they were originally from, were also included. As a dominant social group in China, Han consisted of 94% of the sample size in the present study. The descriptive statistics showed that most students were from rural areas, accounting for 53%. Scott et al. found that for youth students who experienced Hurricane Katrina, their aggressive behaviors were negatively associated with academic performance (18). Similarly, students with a higher academic performance may have better responses to COVID-19. Thus, we include students' academic performance in the models, which was measured by students' GPA on a five-point scale.

### Analytic Plan

Given that the dependent variables were either ordinal or binary variables, the research questions were addressed by generalized linear regression models using Stata 16 software. Before conducting the regression models, multiple imputation was implemented. Here, 10 copies of the data were established, each of which was imputed with a suitable missing value and analyzed independently to make the results more precise (19). The reported parameter estimates were the average of these 10 copies of imputed data.

As these ordinal variables were normally distributed, we treated them as continuous variables for straightforward interpretation in the regression models guided by Pasta (20). First, multiple analysis of variance (MANOVA) was conducted to test whether or not family characteristics–family income, father's occupation, and mother's occupation respectively explain statistically significant variances on multiple continuous dependent variables–awareness, knowledge and preparedness. The statistical indices in Table 2 suggest a statistically significant difference among family income groups, high-income, middle-income, and low-income, on the combined dependent variables, for example, Wilks' Λ = 0.977, *F* (2, 1,467) = 1.93, and *p* <0.05. Looking at the father's occupation, Wilks' Λ = 0.962, *F* (3, 1,466) = 2.09, and *p* <0.01. For the mother's occupation, Wilks' Λ = 0.970, *F* (3, 1,466) = 1.66, and *p* <0.05. These results indicated a significant difference among the father's and mother's occupation groups on the combined dependent variables. Then, we moved to the next step–testing each dependent variable using the same set of control variables, respectively.

**Table 2:** Results of MANOVA for dependent variables of COVID-19 awareness, knowledge and preparedness.

			**Hypothesis**	**Error**	
	**Value**	**F**	***df***	***df***	***p***
**Family income**					
Wilks' lambda	0.977*	1.93	2	1,467	0.010
Pillai's trace	0.024*	1.93	2	1,467	0.010
Hotelling trace	0.024*	1.93	2	1,467	0.010
Roy's largest root	0.018**	2.86	2	1,467	0.002
**Father's occupations**					
Wilks' lambda	0.962**	2.09	3	1,466	0.001
Pillai's trace	0.038**	2.09	3	1,466	0.001
Hotelling trace	0.038**	2.09	3	1,466	0.001
Roy's largest root	0.02**	3.25	3	1,466	0.001
**Mother's occupations**					
Wilks' lambda	0.970*	1.66	3	1,466	0.017
Pillai's trace	0.030*	1.66	3	1,466	0.017
Hotelling trace	0.030*	1.66	3	1,466	0.017
Roy's largest root	0.018**	2.86	3	1,466	0.002

* *p <0.05,*

** *p <0.01. N = 1,470*.

The dependent variables were students' risk awareness of COVID-19. The main independent variables were family income and parental occupations, while holding other students' demographic characteristics and academic performance constant. The initial model only included family income and parental occupations as the independent variables and awareness as the dependent variable. Then, we added students' sex, ethnicity, urbanicity, and GPA one by one to examine the relationship between family characteristics and students' COVID-19 awareness. For simplification, we only present the final model as follows:


(1) $$\begin{array}{r} Awareness=\beta_{0}+\beta_{1}*Family_{i}+\beta_{2}*DC_{i}+\beta_{3}*GPA_{i}, \end{array}$$

where *Awareness* is students' risk awareness of COVID-19; *Family*_*i*_ is a vector of student *i*'s family characteristics (i.e., family income and parental occupations); *DC*_*i*_ is a vector of student *i*'s demographic information, such as sex, ethnicity, and urbanicity; and *GPA*_*i*_ is student *i*'s academic performance measured by their GPA.

Similar processes were conducted when analyzing the relationships among students' knowledge, preparedness, and family characteristics. The equations can be provided as follows:


(2) $$\begin{array}{r} Knowledge=\beta_{0}+\beta_{1}*Family_{i}+\beta_{2}*DC_{i}+\beta_{3}*GPA_{i}, \end{array}$$

(3) $$\begin{array}{r} Preparedness=\beta_{0}+\beta_{1}*Family_{i}+\beta_{2}*DC_{i}+\beta_{3}*GPA_{i}, \end{array}$$

where *Knowledge* and *Preparedness* are students' knowledge of and preparedness for COVID-19, respectively.

Moving to students' responses to campus anti-epidemic climate change, and given the dichotomous nature of these survey items, the logistic regression was conducted as follows:


(4) $$\begin{array}{r} {logit\;\left(\frac{P_{response}}{1-P_{response}}\right)}_{i}=\beta_{0}+\beta_{1}Family_{i}+\beta_{2}DC_{i}+\beta_{3}GPA_{i}, \end{array}$$

where *response* signifies whether campus anti-epidemic climate change affects students' corresponding responses.

Last, interaction variables between sex/ethnicity and family income were generated. They were added into the models where there were significant relationships to identify whether the intentional relationships varied by sex and ethnicity (21).

## Results

### Family Income and Students' Risk Awareness of COVID-19

Three models were assessed using different survey items of awareness as dependent variables in Equation (1) to examine the relationships between students' risk awareness of COVID-19 and family characteristics. Table 3 shows the three full models, including students' sex, ethnicity, urbanicity, and academic performance. From Model 1, it was found that holding other variables constant, low-income students' frequency of discussing COVID-19 with classmates was 0.28 lower than that of high-income students. In contrast, that of mid-income students was 0.22 lower than high-income students.

**Table 3:** The relationships between family characteristics and students' COVID-19 awareness.

		**Model 1 (discuss with classmates)**		**Model 2 (discuss with friends)**		**Model 3 (discuss with family)**	
		**β**	**SE**	**β**	**SE**	**β**	**SE**
**Demographic characteristics**							
*Sex*							
	Female	0.06	0.04	0.10*	0.04	0.09*	0.04
*Ethnicity*							
	Han	0.06	0.09	0.06	0.08	0.12	0.08
*Urbanicity*							
	Suburban	−0.08	0.07	−0.08	0.07	−0.09	0.07
	Rural	0.03	0.05	0.02	0.05	0.02	0.05
**Family characteristics**							
*Family income*							
	Low-income	−0.28*	0.11	−0.28**	0.11	−0.30**	0.11
	Mid-income	−0.22*	0.11	−0.26**	0.10	−0.22*	0.10
*Father*'*s job*							
	Father's job-P	0.01	0.09	−0.05	0.09	−0.05	0.09
	Father's job-S	−0.06	0.09	−0.01	0.08	−0.05	0.09
	Father's job-O	−0.01	0.09	−0.04	0.08	−0.03	0.09
*Mother*'*s job*							
	Mother's job-P	0.09	0.10	0.07	0.10	0.08	0.10
	Mother's job-S	0.05	0.09	−0.03	0.09	0.01	0.09
	Mother's job-O	−0.02	0.09	0.01	0.09	−0.04	0.09
*Academic performance*							
	College GPA	−0.03	0.05	−0.03	0.04	−0.01	0.04
	Constant		2.60***		2.52***		2.43***	
	F test		1.21		1.37		1.62	
	Sample *N*		1,470		1,470		1,470	

* *p <0.05,*

** *p <0.01,*

*** *p <0.001. Male, Other, Urban, High-income, Father's job-G, and Mother's job-G are reference groups and are omitted from this table. SE, standard error*.

Looking at the frequency of discussing COVID-19 with friends (Model 2) and holding other variables constant, low-income and mid-income students were 0.28 and 0.26 lower than high-income students, respectively. Similar results were found in Model 3, where students from high-income families were more likely than those from low- or mid-income families to discuss COVID-19 with their families (β = −0.30, *p* <0.01; β = −0.22, *p* <0.05). In addition, neither the father's occupation nor the mother's occupation was significantly associated with students' risk awareness of COVID-19.

### Family Income, Parental Occupations, and Students' Knowledge of COVID-19

Models 4–6 examine the relationships between students' knowledge of COVID-19 and family characteristics using Equation (2). The results from Table 4 reveal that family income remained a good predictor for three outcome variables measuring students' knowledge of COVID-19. In Model 4, when holding other variables constant, high-income students had better knowledge of COVID-19 symptoms compared to low-income students (β = −0.34, *p* <0.01) and mid-income students (β = −0.30, *p* <0.05). Interestingly, students whose mother's job belonged to the other jobs category had lower knowledge of COVID-19 symptoms than those whose mothers were employed by the government (β = −0.21, *p* <0.05).

**Table 4:** The relationships between family characteristics and students' knowledge of COVID-19.

		**Model 4 (symptom)**		**Model 5 (transmission)**		**Model 6 (preventative measures)**	
		**β**	**SE**	**β**	**SE**	**β**	**SE**
**Demographic characteristics**							
*Sex*							
	Female	−0.09	0.05	−0.10*	0.05	−0.05	0.05
*Ethnicity*							
	Han	−0.05	0.10	−0.04	0.10	−0.00	0.10
*Urbanicity*							
	Suburban	−0.12	0.08	−0.13	0.09	−0.09	0.08
	Rural	−0.15**	0.06	−0.19***	0.06	−0.11*	0.06
**Family characteristics**							
*Family income*							
	Low-income	−0.34**	0.13	−0.37**	0.13	−0.35**	0.13
	Mid-income	−0.30*	0.12	−0.26*	0.13	−0.25*	0.12
*Father's job*							
	Father's job-P	0.06	0.11	−0.08	0.11	−0.18	0.11
	Father's job-S	0.03	0.10	−0.08	0.10	−0.23*	0.10
	Father's job-O	−0.01	0.10	−0.13	0.10	−0.20*	0.10
*Mother's Job*							
	Mother's job-P	−0.13	0.12	−0.01	0.12	0.04	0.12
	Mother's job-S	−0.09	0.10	0.04	0.11	0.11	0.10
	Mother's job-O	−0.21*	0.11	−0.06	0.11	−0.03	0.11
*Academic performance*							
	College GPA	0.06	0.05	0.02	0.05	0.03	0.05
	Constant		3.34***		3.59***		3.59***	
	F test		3.29***		3.57***		2.72***	
	Sample *N*		1,470		1,470		1,470	

* *p <0.05,*

** *p <0.01,*

*** *p <0.001. Male, Other, Urban, High-income, Father's job-G, and Mother's job-G are reference groups and are omitted from this table. SE, standard error*.

Concerning the knowledge of COVID-19 transmission, Model 5 determined that compared to high-income students, that of low-income students was 0.37 lower, and that of mid-income students was 0.26 lower when holding other variables constant. Model 6 examined the relationship between the knowledge of COVID-19 preventative measures and family characteristics. Notably, students whose fathers were employed in service-related fields and other fields had significantly lower knowledge of COVID-19 preventative measures than those whose fathers were employed by governments. Additionally, Table 4 also shows that rural students had significantly lower knowledge of COVID-19 than urban students.

### Family Characteristics, Students' Preparedness for COVID-19, and Students' Responses to Campus Climate Change

By conducting Equation (3), Table 5 reveals no significant relationships between students' preparedness for COVID-19 and their family income. However, it was observed that students whose fathers were professionals or experts were less likely to wear masks than those whose fathers were government employees (β = −0.33, *p* <0.01).

**Table 5:** The relationships between family characteristics and students' preparedness for COVID-19.

		**Model 7 (mask)**		**Model 8 (hand sanitizer)**		**Model 9 (social distance)**	
		**β**	**SE**	**β**	**SE**	**β**	**SE**
**Demographic characteristics**							
*Sex*							
	Female	0.17***	0.05	0.05	0.05	0.07	0.05
*Ethnicity*							
	Han	0.15	0.10	0.13	0.10	−0.06	0.10
*Urbanicity*							
	Suburban	−0.06	0.09	−0.04	0.09	−0.07	0.08
	Rural	0.05	0.06	0.02	0.06	−0.03	0.05
**Family characteristics**							
*Family income*							
	Low-income	0.05	0.13	−0.18	0.14	−0.16	0.13
	Mid-income	0.03	0.12	−0.10	0.13	−0.13	0.12
*Father's job*							
	Father's job-P	−0.33**	0.11	−0.11	0.11	−0.05	0.10
	Father's job-S	−0.05	0.10	0.07	0.11	0.02	0.10
	Father's job-O	−0.17	0.10	−0.13	0.11	−0.06	0.10
*Mother's job*							
	Mother's job-P	0.17	0.12	0.20	0.12	0.15	0.11
	Mother's job-S	0.05	0.11	−0.04	0.11	0.04	0.10
	Mother's job-O	0.11	0.11	0.10	0.11	0.09	0.10
*Academic performance*							
	College GPA	0.07	0.05	−0.03	0.05	0.01	0.04
	Constant	1.79***		2.30***		2.35***	
	F test	2.64**		1.26		0.59	
	Sample *N*	1,470		1,470		1,470	

** *p <0.01,*

*** *p <0.001. Male, Other, Urban, High-income, Father's job-G, and Mother's job-G are reference groups and are omitted from this table. SE, standard error*.

Family income seemed to be a weak predictor concerning students' responses to campus climate change. Statistically significant relationships were only found in Models 12 and 13 in Table 6 by performing Equation (4). Compared to high-income students, mid-income students were less likely to maintain social distancing when they perceived loose campus anti-epidemic measures (OR = 0.58, *p* <0.05). Mid-income students were 45% less likely to participate in campus activities than high-income students. In addition, female students were 1.41 times more likely than male students to perceive climate change, but were 25% less likely to maintain social distance and 27% less likely to participate in campus activities when they felt that the campus anti-epidemic measures were loose.

**Table 6:** The relationships between family characteristics and students' responses to campus climate change.

		**Model 10**		**Model 11**		**Model 12**		**Model 13 (participate**	
		**(perceived climate)**		**(mask-wearing)**		**(keep social distance)**		**in campus activities)**	
		**OR**	**SE**	**OR**	**SE**	**OR**	**SE**	**OR**	**SE**
**Demographic characteristics**									
*Sex*									
	Female	1.41*	0.24	0.93	0.10	0.75**	0.08	0.73**	0.08
*Ethnicity*								
	Han	1.35	0.43	0.80	0.18	1.13	0.25	1.27	0.29
*Urbanicity*								
	Suburban	2.68*	1.11	1.18	0.22	1.02	0.19	1.07	0.20
	Rural	0.85	0.17	1.12	0.14	1.14	0.14	0.96	0.12
**Family characteristics**									
*Family income*									
	Low-income	0.83	0.39	0.74	0.22	0.59	0.17	0.62	0.18
	Mid-income	0.98	0.44	0.67	0.19	0.58*	0.16	0.55*	0.15
*Father's job*									
	Father's job-P	0.99	0.36	0.80	0.19	0.73	0.17	0.67	0.16
	Father's job-S	1.54	0.58	0.80	0.18	0.65	0.15	0.83	0.19
	Father's job-O	1.12	0.40	0.72	0.17	0.71	0.16	0.70	0.16
*Mother's job*									
	Mother's job-P	1.06	0.44	1.33	0.35	1.58	0.42	1.36	0.36
	Mother's job-S	0.95	0.36	1.27	0.30	1.42	0.34	1.10	0.26
	Mother's job-O	1.08	0.41	1.45	0.34	1.59	0.38	1.27	0.30
*Academic performance*									
	College GPA	1.04	0.20	0.97	0.10	1.08	0.12	0.96	0.12
	Constant	4.20		2.22		1.01		1.45	
	F test	1.22		0.59		1.38		1.38	
	Sample *N*	1,470		1,470		1,470		1,470	

* *p <0.05,*

** *p <0.01. Male, Other, Urban, High-income, Father's job-G, and Mother's job-G are reference groups and are omitted from this table. OR, odds ratio; SE, standard error*.

### Whether the Relationships Vary by Sex and Ethnicity

**Figure 1:**
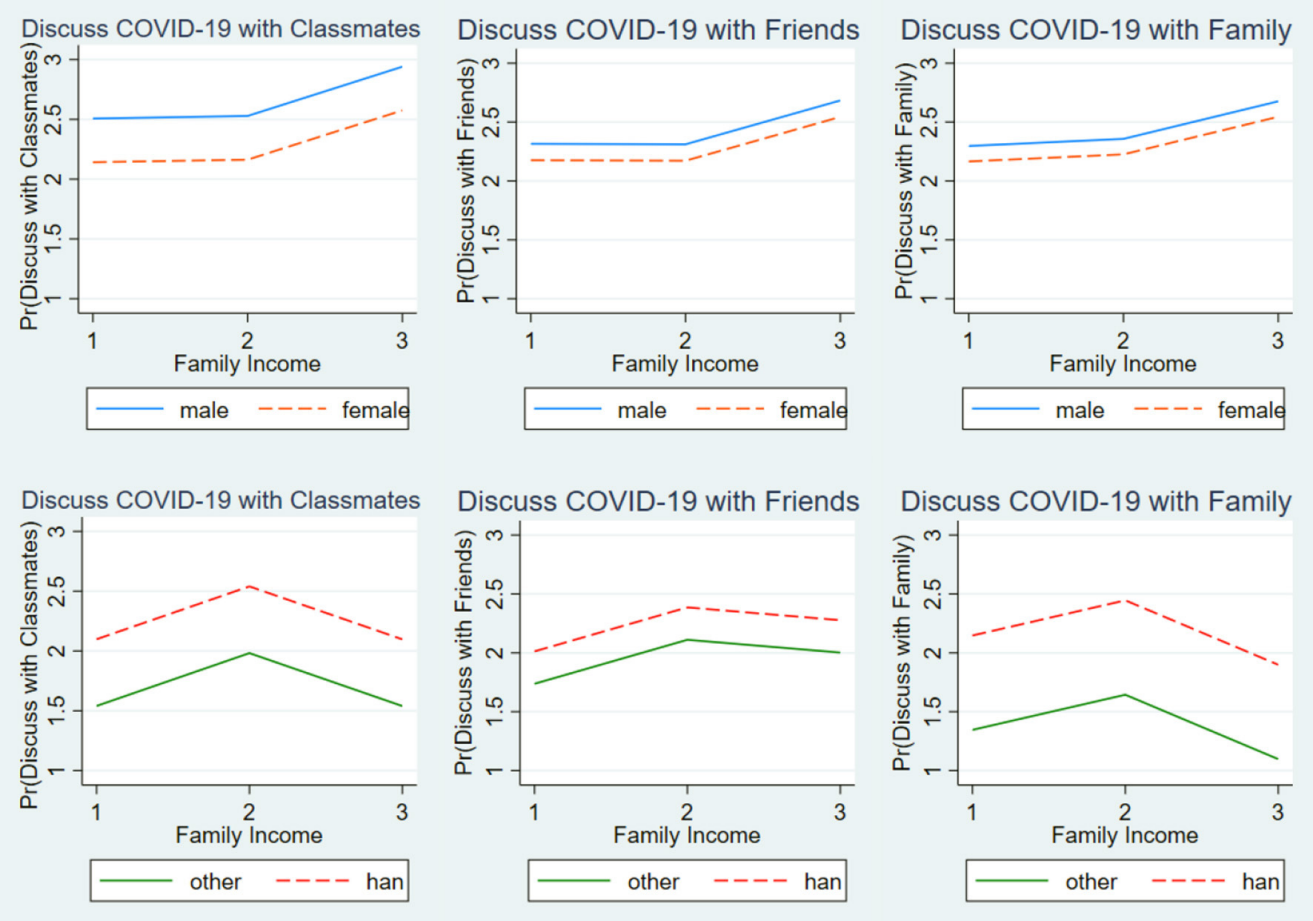
The relationship between family income and students' COVID-19 awareness by sex and ethnicity.

**Figure 2:**
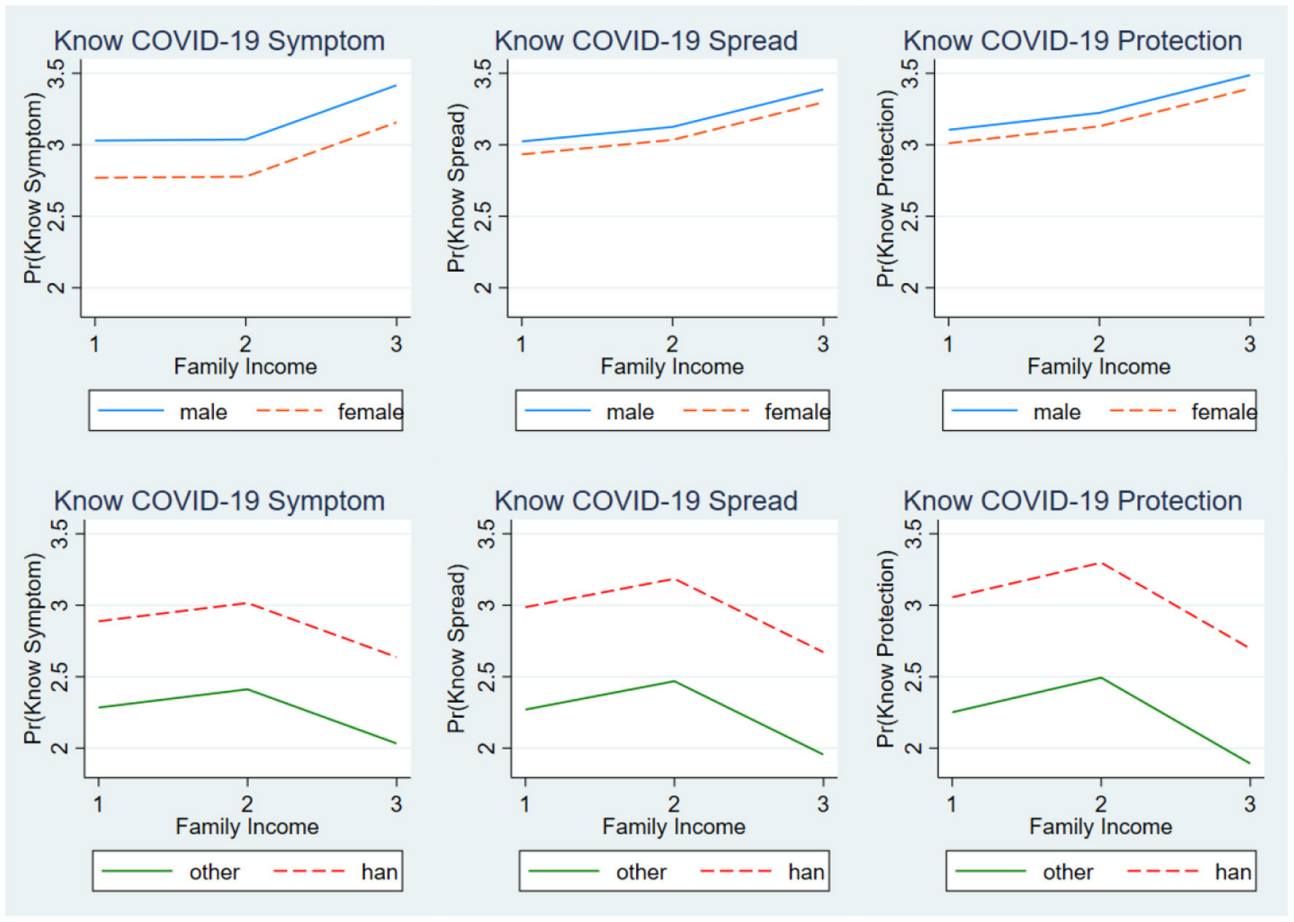
The relationship between family income and students' COVID-19 knowledge by sex and ethnicity.

The present study models were reported with interaction terms using figures for clearer demonstration. Specifically, the postestimation margins command was used to make an estimation from the models with interaction terms. Figure 1 shows whether the relationship between family income and students' COVID-19 awareness varied by sex and ethnicity. Figure 2 depicts whether the relationship between family income and students' COVID-19 knowledge varied by sex and ethnicity. Across the figures, the lines of female and male groups were almost parallel. Furthermore, the lines of Han and other ethnic groups were also almost parallel, indicating that the relationships among family income and students' COVID-19 awareness and knowledge did not significantly differ between female and male students nor between that of Han students and students of other ethnicities.

## Discussion

### Family Characteristics Matter on Students' Responses to COVID-19

This study sought to better understand the roles of family characteristics on students' responses to COVID-19 from the aspects of awareness, knowledge, and preparedness. Family characteristics have been found to impact individuals' disaster responses. For example, Wu et al. surveyed 327 households that experienced the Wenchuan and Lushan earthquakes in China, finding that family characteristics such as annual household income and education level were significantly correlated with residents' disaster knowledge and disaster avoidance behavior (22). Wei et al. found similar results where lower family income and education levels would result in more insufficient disaster response capabilities and more vulnerability (23). In the scenario of COVID-19, we found that students' family characteristics, such as annual family income and parental occupation, contribute to students' awareness and knowledge of COVID-19. Additionally, it was determined that students from wealthier families were more sensitive to campus anti-epidemic climate change. Furthermore, sex was significantly associated with students' responses to COVID-19, whereas ethnicity was not.

The COVID-19 pandemic has significantly influenced the global higher education community (24, 25). Aristovnik et al. found that the pandemic has lowered students' satisfaction with academic work and life worldwide, especially for students with low living standards (26). Thus, exploring the key factors leading to students' appropriate responses to COVID-19, which ultimately facilitates students' college success, is crucial. Our results showed that family income is statistically significant and positively predicted students' risk awareness of COVID-19. This was consistent with the study conducted by Zhang and Qian (27), which found that high-income respondents had a higher awareness of disaster insurance. It is plausible that at high levels of income, individuals with high-risk awareness can pay more toward avoiding or mitigating risks. McDaniels et al. found that household income was strongly associated with a household's willingness to pay for risk reductions (28). Zhou et al. observed that high-income farmers tended to be risk-averse and had a higher risk perception of soil pollution (29). As such, students from wealthier families may be alerted earlier by their parents, and thus exhibit a more heightened risk awareness of COVID-19.

Studies have found that parental education strongly predicts family income (30, 31). Generally, at least one parent is well-educated if the household income is relatively high. It is likely that well-educated parents may have a wide range of knowledge, such as pandemic knowledge, and information-seeking skills (32). They may also have more opportunities to receive information (33). Moreover, wealthier families may access additional resources to reinforce their disaster knowledge. Chan et al. found that in Hong Kong, respondents with higher household income were more likely to receive first aid training (34). In another study, Nipa et al. found that students from high-income families were more willing to take disaster risk reduction courses (35). These factors will increase individuals' disaster-related knowledge. Consistent with these previous studies, the results of the present study have also revealed that students from high-income families were more likely to have a better understanding of COVID-19.

Regarding parental occupation, our results indicated that parental occupations were significantly associated with students' knowledge of and preparedness for COVID-19. These were consistent with Teo et al. (32), which showed that professional or administrative occupations were more likely to have reliable disaster information resources than laborers, machinery operators and drivers, and unemployed individuals. Furthermore, different occupations may have significant differences in acquiring social support. Wu et al. found that government employees' social support was the highest and was prominently higher than those of the unemployed during the COVID-19 pandemic (36). This could help contribute to both the mental and material preparation of the pandemic. Additionally, in the Chinese context, government employees need to play an exemplary role in preventing and preparing for the pandemic. Their general responsibilities include, but are not limited to, being familiar with pandemic-related knowledge, such as what the symptoms of the COVID-19 virus are, how to prevent COVID-19 infection, and informing those around them to be highly aware of and well-prepared for the pandemic. As a consequence, students whose parents were employed by the government were likely to have better knowledge and preparedness.

Lastly, our finding showed that family income seemed to be a weak predictor for students' COVID-19 preparedness. This is perhaps because COVID-19 prevention supplies and materials such as masks and hand sanitizer are not expensive; the COVID-19 tests and vaccines, which are covered by government finances, are free; and, even if individuals have COVID-19, the government will pay for all the treatments. Additionally, an individual's sex seems to be a good predictor in predicting awareness, preparedness, and responses to campus anti-epidemic climate change, which is partially consistent with Zhao et al., observing that girls had higher awareness and better preparedness than boys (37). Perhaps this may be because women are aware that they are in nature physically weaker than men and thus have higher intentions to avoid high risks (38), leading them to be more sensitive to campus anti-epidemic climate change, and avoid campus activities that may have large public gatherings.

The results of this study contribute to the existing literature and have important practical implications. While previous literature has focused on the effects of COVID-19 on family (39–42), this study shifts the lens of how family characteristics influence students' responses to COVID-19. In addition, previous research has explored individual's COVID-19 awareness, knowledge, preparedness, and responses in different contexts with different populations. For example, Al-Hanawi et al. studied general citizens' knowledge, attitude, and practice toward COVID-19 in the Kingdom of Saudi Arabia (43). Iorfa et al. focused on Nigerians' COVID-19 knowledge, risk perception, and precautionary behavior (44). Olaimat et al. investigated university students' COVID-19 knowledge and information seeking in Jordan (45). It is pivotal that our study expands upon these contexts and populations to Chinese undergraduate students because China has a large number of undergraduate students in universities and colleges, which could be a great threat for disease transmission.

For practical implications, understanding the roles of family characteristics on students' responses to COVID-19 is crucial, especially for these vulnerable students. As illuminated in the obtained results, students from low-income and mid-income families, or whose parents were not government employees, experienced disadvantages in COVID-19 awareness and knowledge, as well as responses to campus anti-epidemic climate change. These could help post-secondary administrators make informed decisions on educating socially-disadvantaged students. Activities such as COVID-19 training camps and/or lectures could be offered to these students. School counselors could target disadvantaged students to strengthen their mental health and increase their awareness and knowledge of COVID-19. Governmental organizations such as local health commissions and education departments could disseminate health information targeting low-income families by using social media, texts, or phone calls. All these could help individuals appropriately prepare for a pandemic, ultimately facilitating their college success. Additionally, our results may be generalized to similar post-secondary education settings in other provinces or countries, and could be used to inform corresponding administrators, policymakers, and scholars of the important roles that family characteristics play in response to COVID-19 or other strong infectious diseases similar to COVID-19.

### Limitations and Future Directions

Although the questionnaire gathered abundant information, some other family characteristics were not collected. Other family-related factors, such as parental reactions and interactions within the family, have been found to influence children's disaster reactions (46), which did not appear in the present study. Also, one of the components of family socioeconomics, namely, parental education, was not collected. As noted earlier, parental education may be highly correlated with family income, though it is still worth including in the models. Future research could redesign the surveys and obtain more information on students' families to more comprehensively investigate the effects of family-related factors on students' COVID-19 responses.

Another limitation is that this study could not conclude a causal relationship between family characteristics and students' responses to COVID-19. Evidence-based administrations are interested in drawing causal inferences (47). However, conditions to make causal inferences, such as using a specific dataset and conducting experimental and/or quasi-experimental designs, are required. In some circumstances, for postsecondary stakeholders, it is still valuable to note that the intended relationships indeed exist and are, therefore, needed to make an informed decision.

## Conclusion

Understanding the factors that potentially affect students' COVID-19 responses, such as risk awareness, knowledge of the disease, and pandemic preparedness, is crucial. This study explores the relationships between students' family characteristics and their responses to COVID-19. We found that family income and parental occupations contributed to students' awareness and knowledge of COVID-19. Moreover, female students were more likely to perceive campus anti-epidemic climate change than male students. None of the relationships were found to be varied by either sex or ethnicity. These valued results could help postsecondary administrators take informed actions, which could help vulnerable students appropriately respond to the pandemic, eventually preventing students from being negatively affected by the pandemic and enabling them to achieve college success.

## Data Availability Statement

The data used to support the findings of this study are available from Zhejiang Academy of Higher Education, Hangzhou Dianzi University. Reasonable requests for CSEPPCE data can be made by email: zhaoteng@hdu.edu.cn.

## Ethics Statement

The studies involving human participants were reviewed and approved by Hangzhou Dianzi University. The participants provided their written informed consent to participate in this study.

## Author Contributions

TZ and QS conceptualized the manuscript. QS performed data collection. TZ took on the leading role in cleaning and analyzing the data and in writing the original draft. XH provided editing. All authors have read and agreed to the published version of the manuscript.

## Funding

This research was supported by the Zhejiang Province Association of Higher Education Foundation (KT2021394), the National Social Science Foundation of China (20BGL273, BIA190198), the Fundamental Research Funds for the Provincial Universities of Zhejiang, Hangzhou Dianzi University (GK219909299001-242), and the Research Start-up Fund, Hangzhou Dianzi University (KYS265621012).

## Conflict of Interest

The authors declare that the research was conducted in the absence of any commercial or financial relationships that could be construed as a potential conflict of interest.

## Publisher's Note

All claims expressed in this article are solely those of the authors and do not necessarily represent those of their affiliated organizations, or those of the publisher, the editors and the reviewers. Any product that may be evaluated in this article, or claim that may be made by its manufacturer, is not guaranteed or endorsed by the publisher.
